# Physical activity interventions for post-stroke cognitive recovery: a systematic review and network meta-analysis of comparative effects

**DOI:** 10.3389/fneur.2025.1646328

**Published:** 2025-09-01

**Authors:** Hongyu Wang, Dong Li, Shuang Li, Xiaolin Zhang, Wanli Zang, Ying Zhu, Shixuan Zhang, Feng Xu, Zixian Xiao, Kelei Guo

**Affiliations:** ^1^School of Physical Education and Health, Guangxi Normal University, Guilin, China; ^2^School of Physical Education and Health, Zhaoqing University, Zhaoqing, China; ^3^Postgraduate School, University of Harbin Sport, Harbin, China; ^4^School of Computer Science, Guangxi Normal University, Guilin, China; ^5^College of Physical Education and Health, Guangxi Medical University, Nanning, China

**Keywords:** physical activity, stroke patients, cognitive function, interventions, network meta-analysis

## Abstract

**Background:**

Post-stroke cognitive dysfunction imposes significant burdens on individuals and healthcare systems. Although physical activity are increasingly recognized as adjunct therapies for cognitive rehabilitation, uncertainties persist regarding their comparative effectiveness. The current evidence lacks direct or indirect comparisons of physical activity programs. This study systematically evaluated the effectiveness of intervention measures through network meta-analysis, providing reference measures for cognitive function recovery in stroke populations.

**Methods:**

We systematically searched PubMed, Cochrane Library, Embase, and Web of Science from their inception through August 2024 to identify randomized controlled trials investigating the effects of physical activity interventions on cognitive function in stroke patients. Two independent reviewers conducted literature screening, data extraction, and quality assessment. Network meta-analysis was performed using Stata 15.1.

**Results:**

A total of 26 randomized controlled trials involving 1,408 participants were included in the analysis. The findings revealed that compared with routine medical care, multi-modal exercise significantly improved cognitive function (SMD = −5.58, 95% CI: −8.00 to −3.16), followed by aerobic exercise (SMD = −4.22, 95% CI: −7.04 to −1.41). The surface under the cumulative ranking curve (SUCRA) probabilities for the eight intervention types were as follows: multi-modal exercise (96.7%), aerobic exercise (80.9%), etc.

**Conclusion:**

Our study indicates that multi-modal exercise (e.g., combined programs integrating strength training, balance exercises, and aerobic training such as running and cycling) and high-intensity aerobic exercise show superior efficacy in enhancing cognitive recovery among stroke patients. Furthermore, while physical activity is proven to be beneficial, the major challenge remains in developing effective strategies to promote long-term adherence to regular physical activity routines.

**Systematic review registration:**

https://www.crd.york.ac.uk/prospero/, identifier CRD42024579294.

## 1 Introduction

Stroke is an acute, focal neurological impairment resulting from injury to the blood vessels of the central nervous system (either ischemia or hemorrhage) (1). This condition arises from the sudden rupture or blockage of blood vessels, leading to an interruption of blood flow to the brain, resulting in neurological deficits (2, 3). Stroke constitutes a major global health challenge, ranking as the second leading cause of death after ischemic heart disease, and it is also the third primary cause of disability.

Approximately 25% of adults face the risk of experiencing a stroke (4). Global epidemiological studies estimate that approximately 68.2 million ischemic strokes and 18.9 million hemorrhagic strokes occur annually (5). Stroke severely impacts cerebrovascular health, and if not treated promptly, it may progress to vascular dementia (6). The incidence of stroke remains stable in affluent nations but is increasing in low and middle income economies. Stroke incidence generally increases with age, and existing evidence suggests that even among younger age groups, stroke incidence is on the rise, especially in men, with approximately 10% of strokes occurring in individuals under the age of 50 (7). As a major cause of both long term disability and premature mortality worldwide, stroke imposes substantial challenges on patients, affecting not only their physical and mental well being but also significantly diminishing their overall quality of life (8, 9).

Identifying effective interventions to enhance cognitive function in stroke patients is of considerable clinical and societal importance. Cognitive function encompasses an individual’s ability to acquire, process, store, and apply information, involving various domains, including attention, memory, executive functions, and language skills (10). Studies have demonstrated that cognitive dysfunction is a prevalent complication among stroke survivors, with incidence rates varying between 50 and 70% (11). Within the first year after a stroke, the prevalence of cognitive impairment can reach as high as 38%, largely attributed to declines in brain functions, particularly those related to memory, comprehension, and attention (12, 13). Stroke can severely damage the cerebrovascular health of patients, and if not treated promptly, it may progress to vascular dementia. Cognitive dysfunction not only affects patients’ recovery outcomes and quality of life but also increases the risk of recurrent stroke (14).

Both pharmacological and non-pharmacological treatments offer distinct advantages and characteristics in the recovery of cognitive function in stroke patients (15). Pharmacological treatments work by directly targeting the nervous system, such as the use of cholinesterase inhibitors like donepezil and galantamine, which improve cognitive function and daily living abilities (16). Memantine, due to its favorable safety profile and tolerability, may be effective in treating post-stroke aphasia (17). Additionally, antidepressants such as selective serotonin reuptake inhibitors are commonly used to treat post-stroke depression (18). However, pharmacological treatments may be associated with side effects. This requires a personalized evaluation of the patient’s clinical advantages and possible risks. The drawbacks of pharmacotherapy include adverse effects such as gastrointestinal issues, neurological problems, and allergic reactions (19). Furthermore, long term use of medications may lead to dependency, tolerance, as well as potential teratogenic and carcinogenic effects (20).

Non-pharmacological treatments involve various approaches, such as psychotherapy, cognitive training, acupuncture, hyperbaric oxygen therapy, and repetitive transcranial magnetic stimulation (17). Notably, therapeutic methods like cognitive behavioral therapy and interpersonal psychotherapy have been shown to effectively improve cognitive function in individuals recovering from a stroke (21), with effects potentially lasting for 6 to 12 months. Acupuncture activates neural cells by stimulating sensory receptors, and excites the cognitive regions of the cerebral cortex, while simultaneously inhibiting neuroinflammatory responses and improving brain tissue perfusion (22). Hyperbaric oxygen therapy improves ischemic hypoxic brain injury, induces the formation of new cerebral blood vessels, and alleviates the severity of cognitive impairments (23).

As a crucial component of non-pharmacological treatment, physical activity interventions significantly enhance cognitive rehabilitation outcomes in stroke patients by increasing serum brain-derived neurotrophic factor (BDNF) levels and improving neurological deficits (24). They promote angiogenesis in specific brain regions, enhance cerebral blood circulation and flow, and trigger neurobiological responses that nourish brain cells and clear metabolic waste or *β*-amyloid plaques, thereby reducing risks of mild cognitive impairment and Alzheimer’s disease (25). These interventions, including multi-modal exercise, aerobic exercise, and resistance exercise, can be personalized to individual patient conditions. By increasing BDNF levels, they protect brain tissue, improve post-stroke muscle spasticity, enhance motor function and activities of daily living—key aspects of rehabilitation—playing a vital role in facilitating cognitive recovery (26, 27).

Essential in post-stroke rehabilitation due to their affordability and ease of implementation (28), physical activity interventions have gained widespread acceptance as adjunct therapies. They contribute to overall health and recovery, enhance physical function and rehabilitation outcomes, and reduce complications like cardiovascular diseases, muscle atrophy, joint stiffness, and deep vein thrombosis (29). Furthermore, they improve psychological health by alleviating depression and anxiety, enhance quality of life, increase self-care abilities, and facilitate reintegration into social and family life (30). The American Heart Association and American Stroke Association recommend moderate-intensity physical activity for stroke patients, emphasizing its crucial role in promoting rehabilitation and functional independence.

Previous studies have demonstrated that physical activity interventions can substantially enhance cognitive function in stroke survivors, particularly in domains such as memory, attention, and executive function. Among these interventions, aerobic exercises—including walking, cycling, tai chi, and yoga—have been found to be particularly effective for improving cognitive performance (31). In addition, resistance exercise, which primarily involves muscle coordination, has also been demonstrated to positively influence cognitive function. Moderate intensity aerobic exercise is considered the most promising approach for enhancing overall cognitive function in patients with ischemic cerebrovascular disease (32). The frequency and duration of exercise training are also crucial for improving cognitive function. It is recommended that the optimal exercise frequency should be 2–3 sessions per week, with each session lasting at least 30 min (33). Furthermore, the timing of the exercise intervention and its duration also have a significant impact on cognitive improvement. Starting exercise training within 24 to 48 h after a stroke may be most effective (32, 33).

Previous studies have extensively investigated the association between physical activity and cognitive function in stroke survivors (34–37). While these studies confirm the general benefits of physical activity, conventional meta-analyses in this field are limited to direct comparisons between specific interventions and controls, failing to synthesize evidence across multiple intervention types. This critical gap means they cannot address the relative effectiveness of diverse physical activity regimens, leaving uncertainties about which modalities work best. To address this limitation, our study conducts the first systematic review and network meta-analysis focused specifically on stroke patients, integrating both direct and indirect evidence from high-quality randomized controlled trials. By comparing and ranking different physical activity interventions, we aim to provide a reference for enhancing cognitive function in this population and offer evidence-based recommendations to improve their cognitive recovery.

## 2 Methods

### 2.1 Protocol and registration

This study adhered to the 2020 guidelines set forth by the Preferred Reporting Items for Systematic Reviews and Meta-Analyses (PRISMA) (38), which provided a structured framework for the selection of relevant literature, data organization, statistical evaluation, and the presentation of findings. Furthermore, the study has been registered in the PROSPERO (CRD 42024579294) database.

### 2.2 Data sources and search strategy

We conducted a comprehensive literature review to investigate the relationship between motor and cognitive function in stroke patients by utilizing four electronic databases: PubMed, Cochrane, Embase, and Web of Science. The search utilized a combination of keywords and MeSH terms, including “physical activity,” “stroke,” and “cognitive function.” The initial search encompassed the period from the inception of each database up to August 15, 2024. Furthermore, we carried out a manual review of the reference lists from the systematic reviews and meta-analyses that we identified, as well as those from the studies selected for inclusion in our review. During the screening process, we utilized EndNote X9 a reference management software—to facilitate the organization, deduplication, and tracking of references. This tool helped streamline the handling of large volumes of literature, ensuring that duplicate records were systematically identified and removed before the manual review stage, thereby enhancing the efficiency and accuracy of the screening process.

Following the PICOS principles, the search terms included “Exercises” or “Physical Activity” or “Activities, Physical” or “Physical Activities” or “Exercise, Physical” or “Physical Exercise” OR “Stroke” or “Cerebrovascular Accident” or “Cerebrovascular Accidents” or “CVA (Cerebrovascular Accident)” or “CVAs (Cerebrovascular Accident)” or “Cognitive Function” or “Attention” or “Orientation” or “Memory” or “Executive Functions” or “Language” or “Randomized controlled trial” or “controlled clinical trial” or “randomized” or “placebo” or “randomly.” For detailed search strategies, please consult Appendices A1, A2.

### 2.3 Study selection and eligibility criteria

This systematic review, based on the PICOS framework, established criteria for the selection, inclusion, and exclusion of literature.

Inclusion criteria for literature were as follows:

(1) Adherence to PRISMA guidelines, with prioritized inclusion of randomized controlled trials to ensure study quality and control for biases.
(2) Diagnosis of stroke must be confirmed according to criteria from the World Health Organization or authoritative clinical guidelines (e.g., AHA/ASA).
(3) Physical activity interventions based on quantifiable metrics
(4) Control group: Participants received active controls or standard treatment/care.
(5) Report of quantitative cognitive outcomes in stroke patients pre- and post-intervention, with validated cognitive assessment tools providing baseline and follow-up data.
(6) Extractable raw data or statistically derivable parameters for subsequent analysis.
(7) Full-text articles published in peer-reviewed English-language journals only.

(8) Exclusion criteria for literature were as follows:

(1) Studies utilizing non-randomized controlled trial designs or reported as conference abstracts.
(2) Research involving participants with comorbid neurological disorders or reporting non-stroke-specific recovery outcomes.
(3) Interventions lacking physical activity components.
(4) Studies reporting qualitative conclusions only, incomplete statistical parameters, or outcomes assessed via non-validated measurement tools.
(5) Publications in non-English languages, unpublished preprints, or standalone abstracts without full-text peer-reviewed versions.

### 2.4 Data extraction

Data from included trials were independently extracted by two authors (HYW and DL), with discrepancies resolved via group discussions. Extracted information included: (1) descriptive details (authors, year, country); (2) participant characteristics (age range, gender, sample size); (3) intervention parameters (time, frequency, duration); and (4) cognitive function outcomes.

For graphically reported data, Engauge Digitizer 12.1 was used following strict protocols: image calibration with known coordinates, duplicate extraction with consensus resolution, and cross-validation against summary statistics. Only post-intervention data were extracted for consistency. Missing standard deviations (SMD) were imputed from 95% confidence intervals (CIs) using. Studies lacking SD/CI were excluded. The categorization of physical activity interventions was based on the American Physical Activity Guidelines (39), complemented by existing systematic reviews on physical activity intervention classification.

For the categorization of physical activity interventions, we referenced.

information related to exercise classification. We categorized physical activity.

interventions into the following major types (39–43) to compare their effects:

(1) Aerobic exercise, including continuous or low intensity intermittent exercises such as running;
(2) Resistance exercise, including resistance exercise performed by overcoming body weight or applying external resistance;
(3) Mind–Body exercise, involving practices such as Tai Chi, Yoga, Yi Jin Jing, and dance;
(4) Stretching exercise, a static or dynamic exercise designed to enhance muscle control, flexibility, and range of motion;
(5) Sensory-Motor training, an integrated training method designed to enhance physical coordination, balance, and control by improving the function of the sensory and motor systems;
(6) Multi-modal exercise, which combines at least two types of exercises, such as Aerobic Exercise and Resistance Exercise.

Commonly used assessment tools include: Rey Complex Figure Test and Recognition Trial; Montreal Cognitive Assessment; Addenbrooke’s Cognitive Examination-Revised; Mini Mental State Examination; Stroop Task; Trail Making Test; Mental Rotation Test; Forward and Backward Digit Span Tests; Verbal Digit Span Test; Functional Flexibility Measurement; and others.

### 2.5 Methodological quality assessment

The methodologies utilized in the studies included in this analysis were evaluated through the Cochrane Risk of Bias tool, which encompasses an examination of seven essential domains: (1) the generation of random sequences (selection bias), (2) the concealment of allocation (selection bias), (3) the blinding of both participants and personnel (performance bias), (4) the blinding of outcome assessors (detection bias), (5) the management of incomplete outcome data (attrition bias), (6) the occurrence of selective reporting (reporting bias), and (7) other potential sources of bias (24, 44, 45).

### 2.6 Statistical analysis

All cognitive outcome indicators were uniformly converted to Standardized Mean Differences (SMD) with directional adjustment to ensure “higher scores = improved function.” For continuous outcomes, these SMDs (and their 95% Confidence Intervals [CIs]) were calculated to establish comparability across diverse scales automatically relaxing assumptions about differences in measurement tools or populations between studies and to enable pooling of overall effects under a single random-effects model. This model, chosen for its ability to maintain consistency amid scale diversity, was also used to estimate overall differences. To assess statistical heterogeneity, we used the chi-square test *p*-value and I^2^ statistic (I^2^ > 50% = moderate heterogeneity; >75% = high heterogeneity). Importantly, further sensitivity analyses (with sequential exclusion of each study) did not alter effect directions, supporting the aggregation of diverse cognitive endpoints into one analytical framework. Collectively, these results indicate good homogeneity in methodology and effect directions across studies, validating the rationale for overall pooled analysis. In line with PRISMA-NMA guidelines, a frequentist network meta-analysis was conducted using maximum likelihood estimation in Stata 15.1 (46), with its “network” package generating evidence network plots (node size = sample size; line thickness = number of comparative studies). Node-splitting analysis assessed consistency by separating direct (from head-to-head studies) and indirect (via common comparators) evidence, with consistency confirmed if *p* > 0.05. To evaluate effectiveness, SUCRA was calculated by estimating rank probabilities, generating cumulative curves, and computing curve areas (0–100%, higher = more effective). For publication bias, we constructed funnel plots, performed Begg’s and Egger’s tests, and applied the trim-and-fill method with a random-effects model to adjust for bias impacts.

## 3 Results

### 3.1 Trial selection

**Figure 1:**
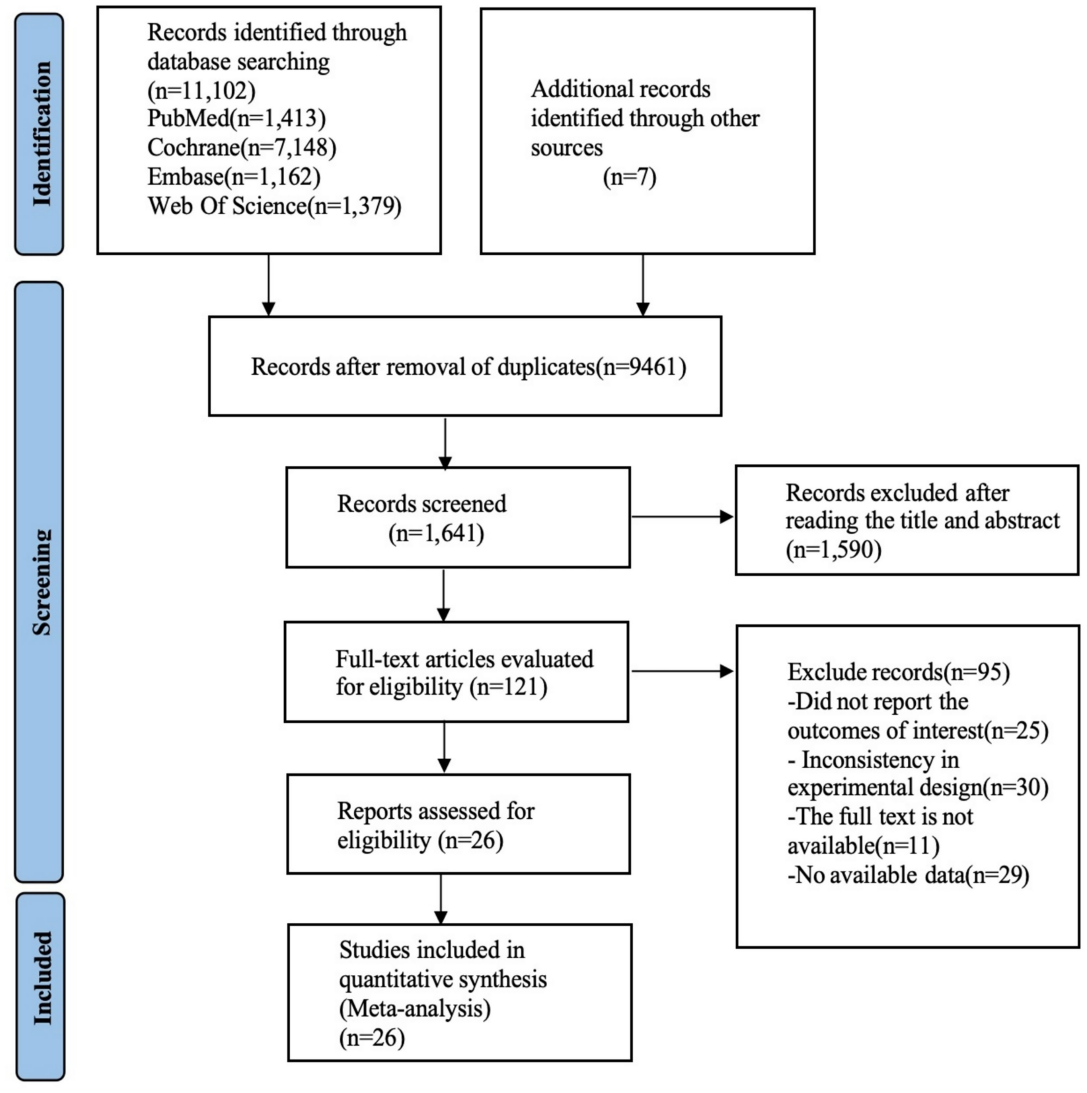
A summary of the evidence searches and selection process.

As depicted in Figure 1, an initial total of 12,048 records was obtained from the database, along with 121 additional records from other sources. Following the removal of duplicates, 9,461 studies remained, After reviewing the titles and abstracts, a total of 64 studies were identified as potentially eligible for inclusion. A full text review led to the exclusion of 38 studies: (1) non-English articles (*n* = 3); (2) study protocols (*n* = 11); (3) conference abstracts (*n* = 5); (4) studies without a control group (*n* = 13); (5) studies with data that could not be extracted (*n* = 6). Finally, 26 studies (47–72) were deemed eligible for systematic review and network meta-analysis.

### 3.2 Trial characteristics

This meta-analysis included 26 studies (Table 1), with a total of 1,408 participants and a mean age of 51.6 ± 6.1 years. These studies were conducted across 12 countries, with 6 studies conducted in China (48, 51, 55, 59, 66, 70), 4 in Canada (56, 60, 63, 65), and 2 each in the United States (53, 64), India (57, 62), South Korea (50, 61), and Turkey (49, 71). Germany (67), Saudi Arabia (54), Denmark (47), Egypt (72), Japan (52), the Netherlands (68), Portugal (58), and the United Kingdom (69) each contributed one study. Among the 26 studies, 9 focused on multi-modal exercise (55, 58, 60, 64, 68–70), 8 on aerobic exercises (47, 49, 55, 56, 58, 63, 65, 72), 8 on mind–body exercise (48, 53, 57, 59, 61, 64, 66, 67), 6 on stretching exercise (47, 54, 62, 63, 69), 5 on sensory-motor training (50, 60, 65, 70, 71) and 3 on resistance exercises (49, 52, 62). Additionally, 21 studies involved control groups that did not receive physical activity interventions. The average intervention duration was 16 ± 7.1 weeks, with 73.08% of studies having an intervention period of 12 weeks or longer. The average frequency of interventions was 32.2 sessions per week, with an average session duration of 513.5 min.

**Table 1:** Summary table of included reviews.

No.	Study	Country	N (IG; CG)	Age (IG; CG)	Intervention (IG)			Intervention (CG)			Population	Outcomes
					Intervention content	Intervention time, Frequency, period	Type	Intervention content	Intervention time, frequency, period	Type		
1	Pallesen et al. (2019) (47)	Denmark	15; 15	55 (22–67); 55 (22–67)	High-Intensity Aerobic Exercise	50 min, 2 weekly, 4 weeks	Multi-modal exercise	Low-intensity aerobic exercise	50 min, 2 weekly, 4 weeks	Aerobic exercise	Stroke patients	RCFT
2	Zheng et al. (2020) (48)	China	22; 19	45–75; 45–75	The Eight Pieces of Brocade	40 min, 3weekly, 24 weeks	Mind–body exercise	Conventional medical care	Maintaining rehabilitation therapy	Routine medical	Stroke patients with cognitive impairment	MoCA
3	Ersoy and Iyigun (2020) (49)	Turkey	20; 20	NA; NA	Virtual boxing	30 min, 3 weekly, 8 weeks	Aerobic exercise	Authentic boxing training	90 min, 1 weekly, 12 weeks	Resistance exercise	Stroke patients	ACE-R
4	Kim and Jang (2021) (50)	South Korea	19:20	NA; NA	Proprioceptive neuromuscular facilitation	30 min, 5 weekly, 6 weeks	Sensory-motor training	Conventional physical therapy	30 min, 5 weekly, 6 weeks	Routine medical	Stroke patients	MMSE
5	Li et al. (2021) (51)	China	100; 100	50–75; 50–75	Finger movement training	2 dayly, 3 months	Stretching exercise	Routine Education and Early rehabilitation Care for Ischemic Stroke	Maintaining rehabilitation therapy	Routine medical	Patients with Ischemic Stroke	MoCA
6	Haruyama et al. (2017) (52)	Japan	16:16	67.56 (10.11); 65.63 (11.97)	Specific Core Stability Training	60 min, 5 weekly, 4 weeks	Resistance exercise	Standard Rehabilitation program	60 min, 5 weekly, 4 weeks	Routine medical	Stroke patients	VST
7	Taylor-Piliae and Coull (2011) (53)	United States	29; 29	>50;>50	Tai Chi	60 min, 3 weekly, 12 weeks	Mind–body exercise	Routine care	Maintaining rehabilitation therapy	Routine medical	Stroke patients	MMSE
8	Aloraini (2022) (54)	Saudi Arabia	19; 19	NA; NA	Lower Limb Constraint-Induced Movement Therapy	210 min, 5 weekly, 2 weeks	Stretching exercise	Standard rehabilitation therapy	270 min, 5 weekly, 2 weeks	Routine medical	Stroke patients	MMSE
9	Yeh et al. (2021) (55)	Taiwan	20; 18	20–90; 20–90	Combining Aerobic exercise with computerized Cognitive training	60 min, 3 weekly, 12 weeks	Multi-modal exercise	Progressive Resistance Stationary Cycling Aerobic Exercise Training/Computer-Based Cognitive Training Group	60 min, 3 weekly, 12 weeks	Aerobic exercise	Stroke patients	MoCA
10	Andrushko et al. (2023) (56)	Canada	14; 11	21–85; 21–85	High-intensity exercise	23 min, 3 daily, 72 days	Aerobic exercise	Watching a documentary	23 min,	Psychotherapy	Stroke patients	MoCA
									3 daily,			
									72 days			
11	Kashyap et al. (2023) (57)	India	40; 40	>18; >18	Yoga qigong	60 min, 4/5 weekly, 3 months	Mind–body exercise	Standard care	Maintaining rehabilitation therapy	Routine medical	Stroke patients	MoCA
12	Maeneja et al. (2023) (58)	Portugal	17; 17	> = 40; > = 40	Running + cycling	45 min, 3 weekly, 12 weeks	Multi-modal exercise	Dual-task gait training group	45 min,	Aerobic exercise	Stroke patients	MMSE
									3 weekly,			
									12 weeks			
13	Xia et al. (2023) (59)	China	35; 35	57.94 (9.38); 58.23 (11.81)	Incorporating Six-Character Mantra Qigong alongside conventional speech therapy	40 min, 5 weekly, 4 weeks	Mind–body exercise	Standard speech therapy	20 min, 5 weekly, 4 weeks	Routine medical	Post-stroke Spastic Dysarthria	MoCA
14	Adjetey et al. (2023) (60)	Canada	34; 52	71; 70	Exercise program	60 min, 2 weekly, 26 weeks	Multi-modal exercise	Balance and Conditioning Program	60 min, 2 weekly, 26 weeks	Routine medical	Patients with Stroke and Severe Cognitive Impairment	MoCA
14	Adjetey et al. (2023) (60)	Canada	34; 52	71; 70	Cognitive and socially enriching activities program	60 min, 2 weekly, 26 weeks	Sensory-motor training	Balance and Conditioning Program	60 min,	Routine medical	Patients with Stroke and Severe Cognitive Impairment	MoCA
									2 weekly,			
									26 weeks			
15	Song et al. (2021) (61)	Korea	18; 16	57.18 (10.65); 58.72 (17.13)	Tai Chi Chuan	50 min, 3 weekly, 3 months	Mind–body exercise	Symptom management	No report	Routine medical	Stroke survivors	MoCA
16	Khurana et al. (2021) (62)	India	10; 10	56.10 (8.07); 56.10 (7.534)	Swiss ball exercises	45 min, 5 weekly, 4 weeks	Resistance exercise	Base exercise	45 min,	Stretching exercise	Subacute stroke patients	MMSE
									5 weekly,			
									4 weeks			
17	Quaney et al. (2009) (63)	Canada	19; 19	64.10 (12.30); 58.96 (14.68)	Aerobic exercise	45 min, 3 weekly, 8 weeks	Aerobic exercise	Stretching exercise	25–30 min,	Stretching exercise	Chronic stroke survivors	ST
									3 weekly,			
									8 weeks			
18	Taylor-Piliae et al. (2010) (64)	United States	37;56	70.6 (5.9); 68.2 (6.2)	Tai Chi Chuan	45 min, 3 weekly, 1 year	Mind–body exercise	Healthy Aging Course	90 min,	Psychotherapy	Stroke patients	F-DST&B-DST
									1 weekly,			
									6 months			
18	Taylor-Piliae et al. (2010) (64)	United States	39;56	68.5 (5); 68.2 (6.2)	Western exercise	45 min, 3 weekly, 1 year	Multi-modal exercise	Healthy Aging Course	90 min,	Psychotherapy	Stroke patients	F-DST&B-DST
									1 weekly,			
									6 months			
19	Tang et al. (2016) (65)	Canada	25;25	62–71; 62–75	High-intensity aerobic exercise	60 min, 3 weekly, 6 months	Aerobic exercise	Low-intensity balance and agility activities	60 min,	Cognitive training	Stroke patients	V-DST
									3 weekly,			
									6 months			
20	Yu et al. (2022) (66)	Hong Kong	10;12	67.3 (4.2); 67.6 (8.1)	Yang-style Tai Chi training	60 min, 3 weekly, 24 weeks	Mind–body exercise	Keep up daily activities	60 min,	Routine medical	Stroke patients	MoCA-HK
									3 weekly,			
									24 weeks			
21	Tripp and Krakow (2013) (67)	Germany	14;16	64.8 (15.0); 65.0 (15.1)	Hydrotherapy	45 min, 3 weekly, 2 weeks	Mind–body exercise	Standard physical therapy	45 min,	Routine medical	Stroke patients	FFM
									2 weekly,			
									2 weeks			
22	Deijle et al. (2023) (68)	Netherlands	60;59	64.2 (9.0); 63.4 (10.5)	Exercise intervention	1 year	Multi-modal exercise	Usual care	No report	Routine medical	Stroke patients	MRT
23	Moore al. (2014) (69)	United Kingdom	20;20	68 (8); 70 (11)	Structured exercise intervention	45–60 min, 3 weekly, 19 weeks	Multi-modal exercise	Family Stretching Program	45–60 min,	Stretching exercise	Stroke patients	ACE-R
									3 weekly,			
									19 weeks			
24	Bo et al. (2019) (70)	China	44;47	66.68 (2.44); 64.36 (2.31)	Combined intervention of physical exercise and cognitive training	3 weekly, 12 weeks	Multi-modal exercise	Routine care	45 min,	Routine medical	Stroke patients	MRT
									3 weekly,			
									12 weeks			
25	Bo et al. (2019) (70)	China	45;47	65.12 (2.56); 64.36 (2.31)	Cognitive training	3 weekly, 12 weeks	Sensory-motor training	Routine care	45 min,	Routine medical	Stroke patients	MRT
									3 weekly,			
									12 weeks			
26	Bo et al. (2019) (70)	China	42;47	67.51 (2.24); 64.36 (2.31)	Physical exercise	3 weekly, 12 weeks	multi-modal exercise	Routine care	45 min,	Routine medical	Stroke patients	MRT
									3 weekly,			
									12 weeks			
27	Ozen et al. (2021) (71)	Turkey	15;15	62.00 (13.12); 69.8 (8.41)	Computer-based exercise	90 min, 5 weekly, 4 weeks	Sensory-motor training	Occupational therapy	60 min,	Routine medical	Stroke patients	MoCA
									5 weekly,			
									4 weeks			
28	El-Tamawy et al. (2012) (72)	Egypt	15;15	49.67 (6.98); 48.4 (6.39)	Stretching exercise	25–30 min, 3 weekly, 8 weeks	Stretching exercise	Aerobic exercise on bicycles	25–30 min,	Aerobic exercise	Stroke patients	ACE-R
									3 weekly,			
									8 weeks			

IG: intervention group; CG: control group; N: Number; NA: not available; RCFT: Rey Complex Figure Test and Recognition Trial; MOCA: Montreal Cognitive Assessment; ACE-R: Addenbrooke’s Cognitive Examination; MMSE: Mini Mental State Examination; ST: Stroop task; MoCA-HK: Hong Kong version of the Montreal Cognitive Assessment; MRT: Mental Rotation Test; F-DST&B-DST: forward and backward digit-span tests; V-DST: Verbal Digit Span Test; FFM: functional flexibility measurement; VST: Victoria Stroop Test.

### 3.3 Risk of bias

**Figure 2:**
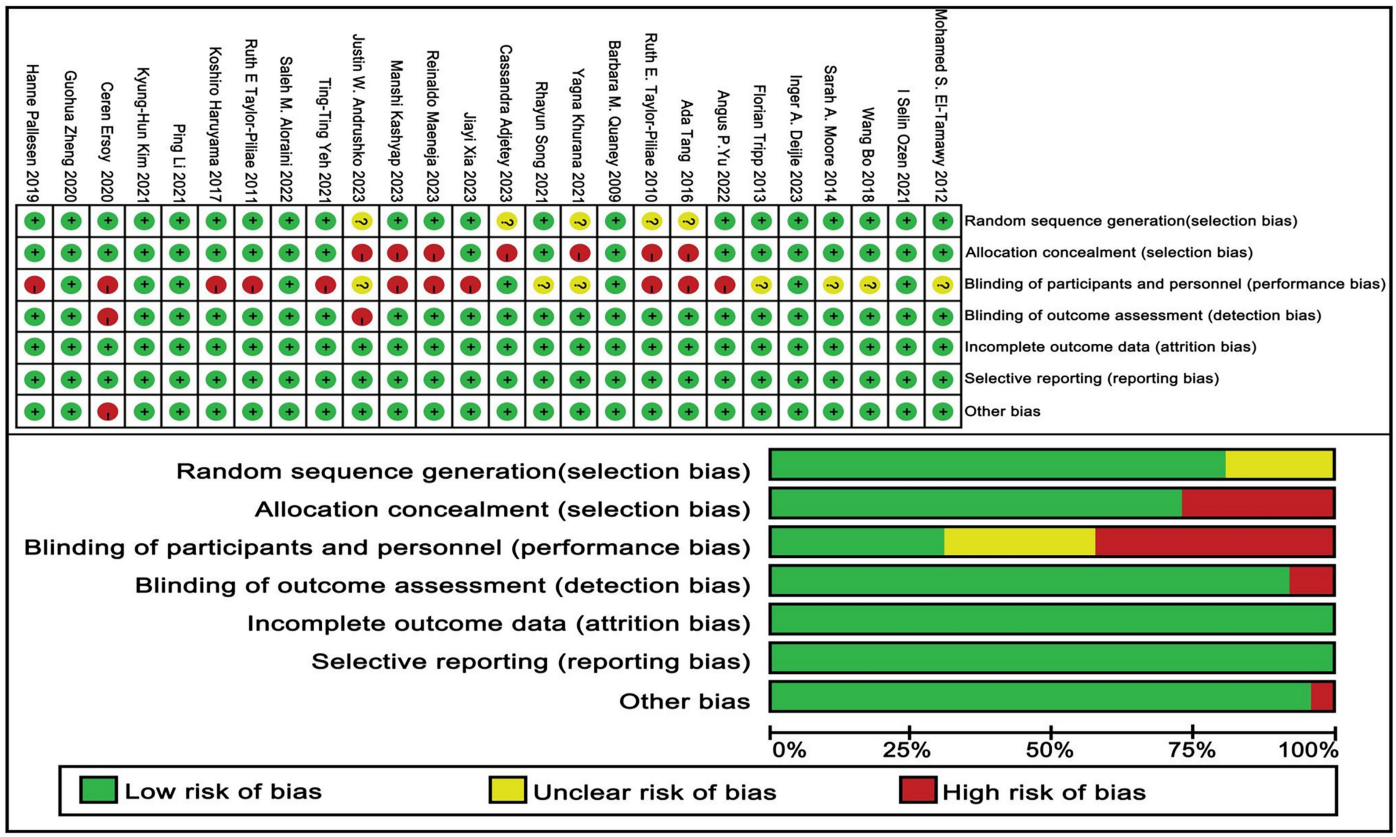
Risk of bias of included studies.

Out of the 26 studies included in the analysis, 21 were found to demonstrate a low risk of bias related to the randomization process, whereas 5 studies were found to lack adequate information regarding their randomization methods. Furthermore, all studies were categorized as exhibiting a low risk of bias concerning deviations from the intended interventions and the management of missing outcome data. In terms of bias risk related to outcome measurement, a total of 24 studies were evaluated and determined to have a low risk, while 2 studies were identified as having a high risk. Regarding selective reporting bias, all 26 studies were assessed to carry a low risk. After a collective evaluation of five specific criteria, the overall risk of bias across the studies was determined. Of the total, 26 studies were classified as having a low overall risk, whereas 14 studies were recognized as exhibiting a high overall risk. A thorough summary of the bias assessment is available in Figure 2 and in Appendix B.

### 3.4 Network meta-analysis

**Figure 3:**
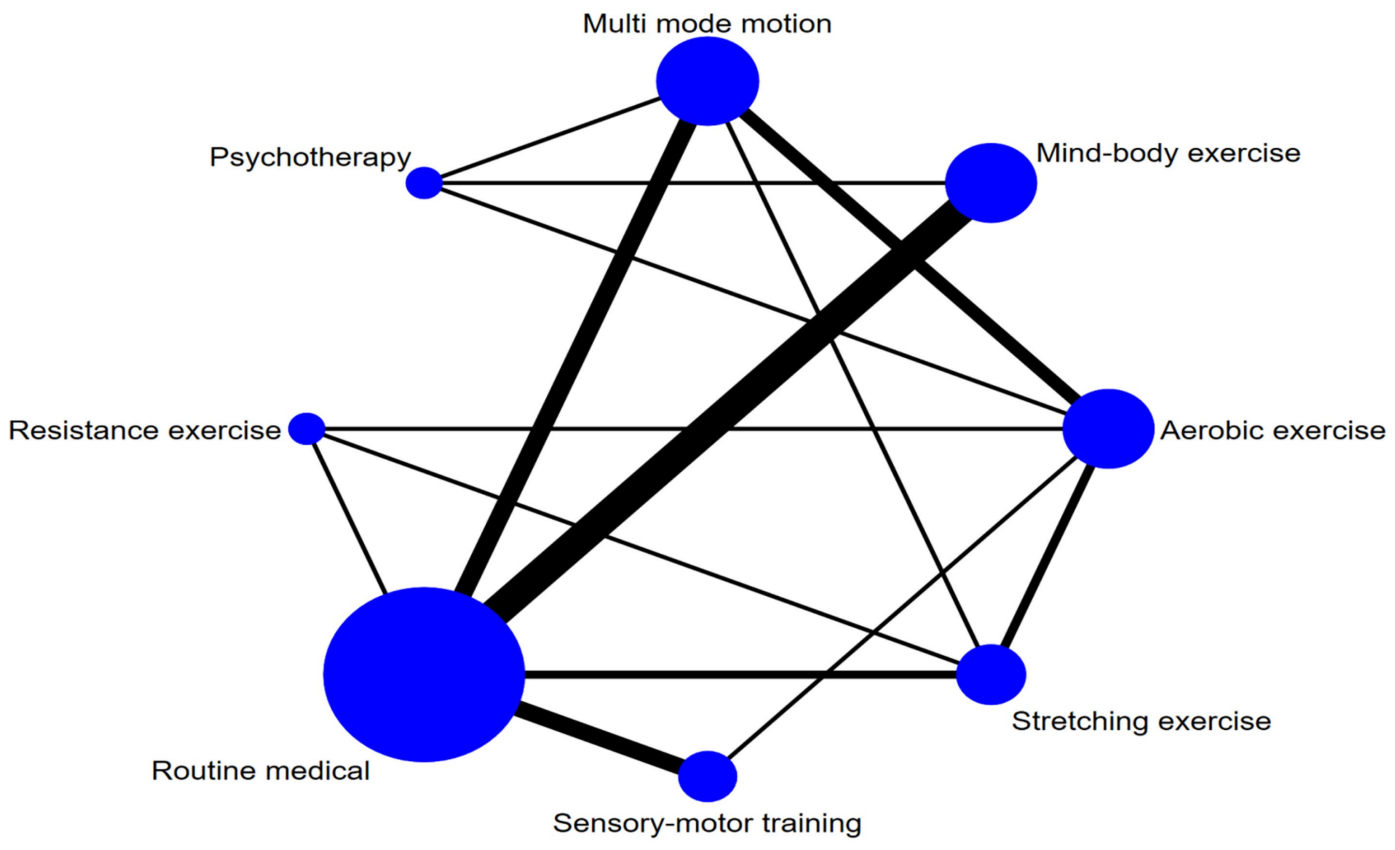
Network diagram.

This study compared the cognitive rehabilitation efficacy of eight post-stroke interventions [multi-modal exercise, aerobic exercise, psychological therapy, resistance exercise, stretching exercise, mind–body exercise, sensorimotor training, and routine medical care] through network meta-analysis (Figure 3). The network structure identified multi-modal exercise (9 studies), aerobic exercise (8 studies), and routine medical care (21 studies) as central nodes, supporting direct comparisons including multi-modal exercise–routine medical care (9 trials), aerobic exercise–routine medical care (8), and mind–body exercise–routine medical care (8). Stretching exercise, resistance exercise, and sensorimotor training relied on indirect evidence due to absent direct comparisons.

Multi-modal exercise showed superior cognitive outcomes: significant improvements over stretching exercise [SMD = 3.49 (95% CI: 0.66, 6.32)], mind–body exercise [SMD = 3.97 (1.17, 6.77)], and sensorimotor training [SMD = 5.74 (2.13, 9.35)] (Table 2). Aerobic exercise demonstrated efficacy against sensorimotor training [SMD = 4.38 (0.58, 8.18)], but its direct comparison with multi-modal exercise was nonsignificant [SMD = −1.36 (−3.82, 1.11)]. Psychological therapy exhibited borderline significance over routine medical care [SMD = −2.93 (−0.30–6.17)].

**Table 2:** League table on interventions.

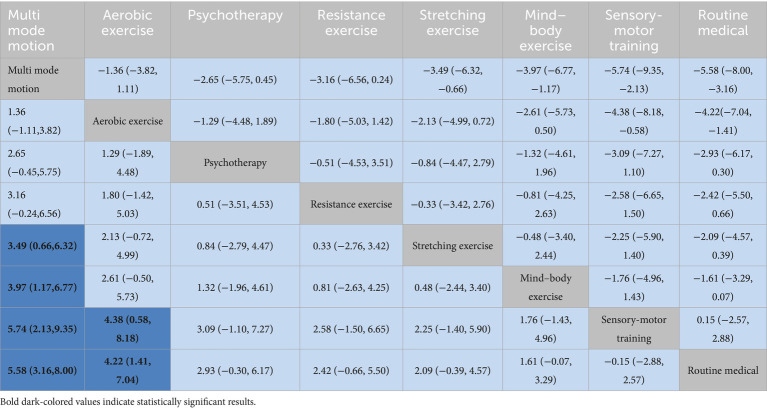

**Figure 4:**
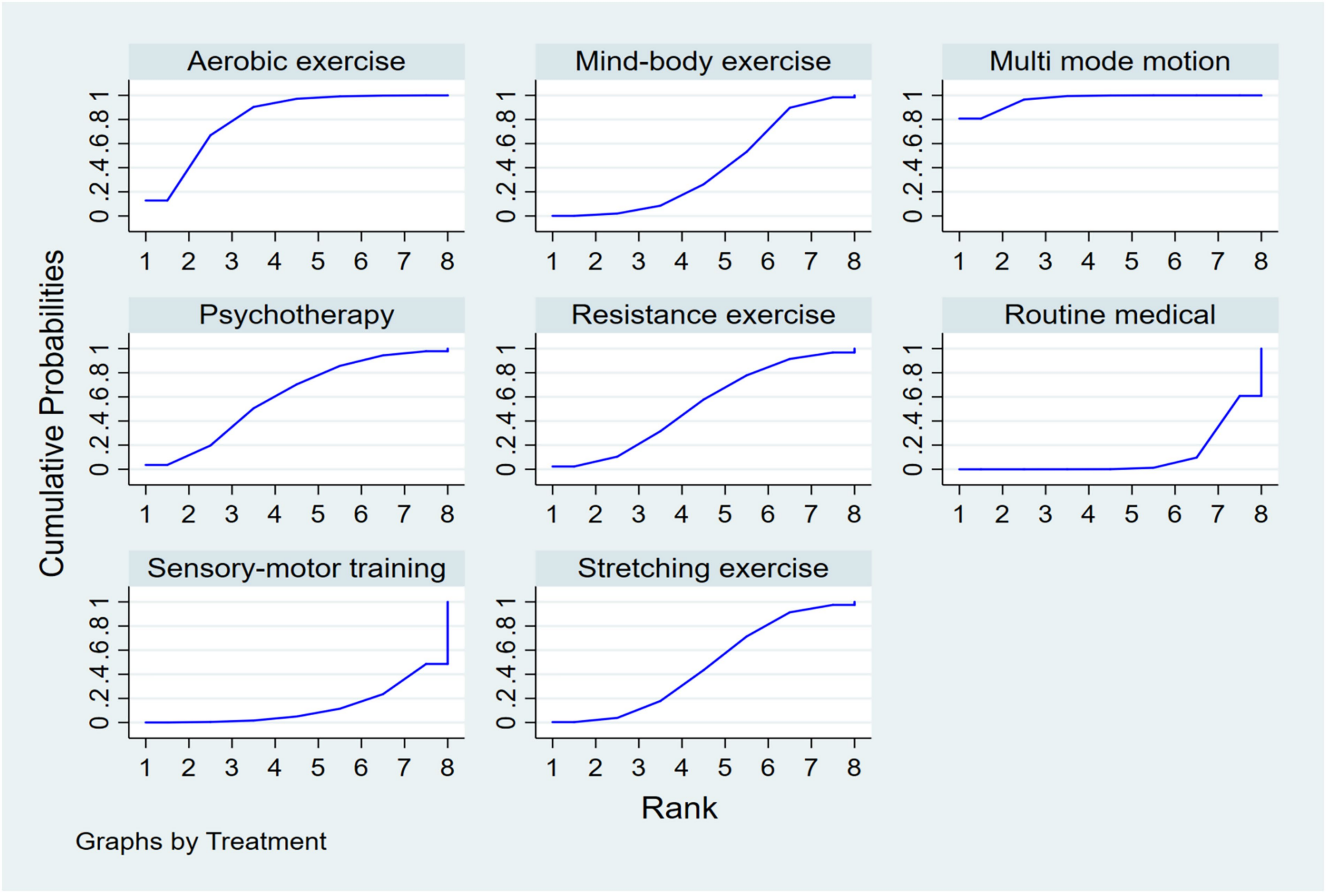
SUCRA plot.

Rankings indicated multi-modal exercise as optimal (SUCRA = 96.7%), followed by aerobic exercise (SUCRA = 80.9%). Sensorimotor training (SUCRA = 13.0%) and routine medical care (SUCRA = 10.3%) ranked lowest (Figure 4).

### 3.5 Publication bias

**Figure 5:**
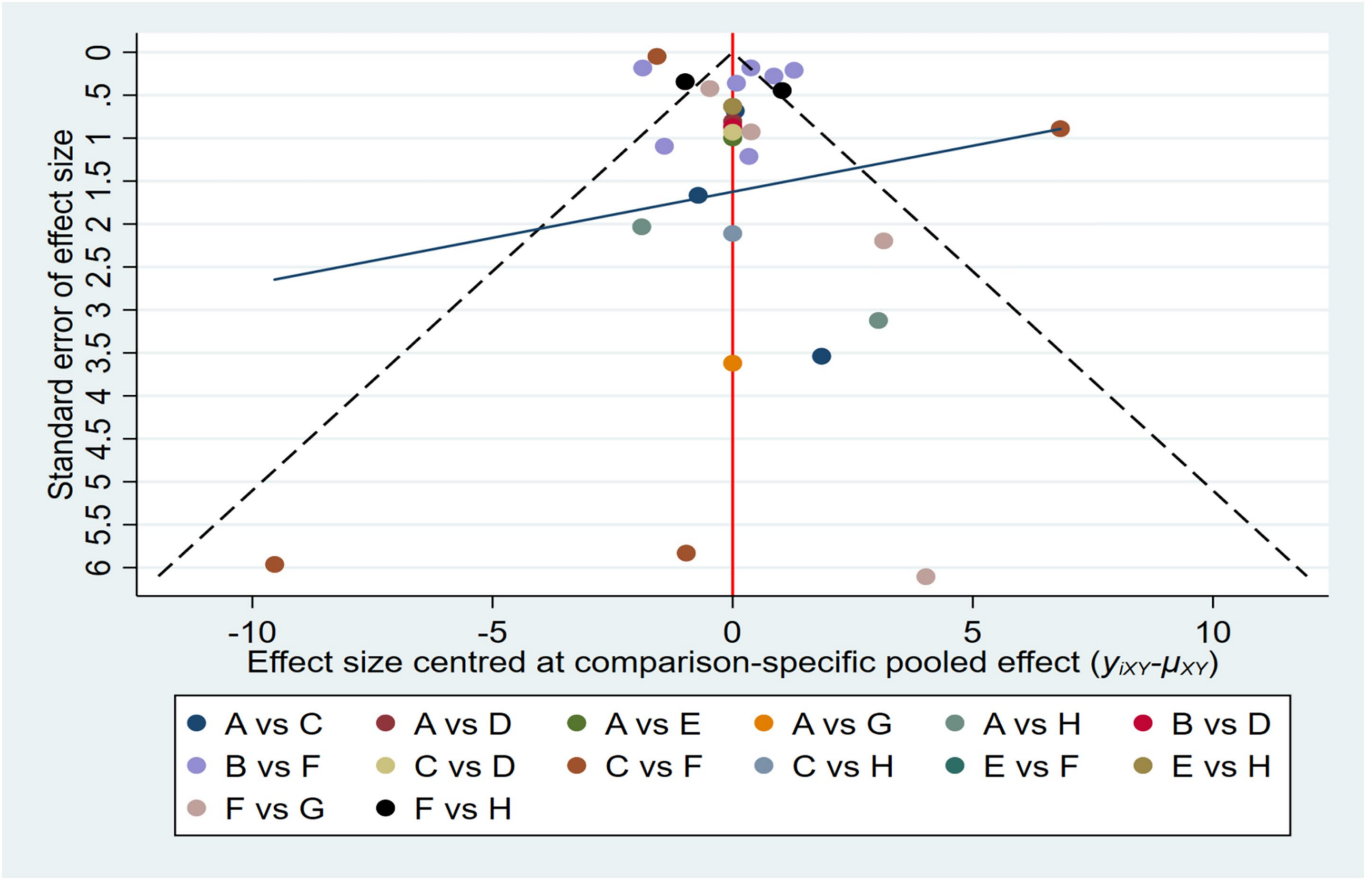
Funnel plot on publication bias.

As illustrated in Figure 5, an initial evaluation of publication bias was performed utilizing a funnel plot. This plot exhibited a generally symmetrical distribution of the studies. This indicates that a visual inspection yielded no clear evidence of publication bias. Following this, We subsequently conducted both the Begg and Egger tests to assess potential bias in the studies. The Begg test revealed no significant bias (z = 1.94, *p* = 0.053), while the Egger test showed significant bias (bias term: t = −0.19, *p* = 0.851), as outlined in Appendices C1, C2. To further explore the potential for publication bias, we carried out a trim and fill analysis utilizing a random effects model. The results indicated a slight reduction in the estimated effect size following the trim and fill adjustment, although the extent of this change was negligible. This suggests that, while it is possible that some degree of publication bias exists, its impact on the overall findings is minimal, its influence on the overall conclusions remains negligible. Notably, the overall effect size remained statistically significant, confirming the robustness of the findings (see Appendices C3, C4). Additionally, a sensitivity analysis, where studies were removed one by one, showed no substantial impact on the overall results (refer to Appendices C5, C6).

## 4 Discussion

This study aims to investigate the comparative impacts of different physical activity interventions on the cognitive functioning of stroke survivors. The results indicate that there are three key interventions aimed at enhancing cognitive function in individuals who have experienced a stroke, in order of effectiveness, are multi-modal exercise, aerobic exercise, and resistance exercise (73). For a more thorough understanding of the outcomes, please refer to the detailed information presented in Table 3.

**Table 3:** Ranking of SUCRA probabilities.

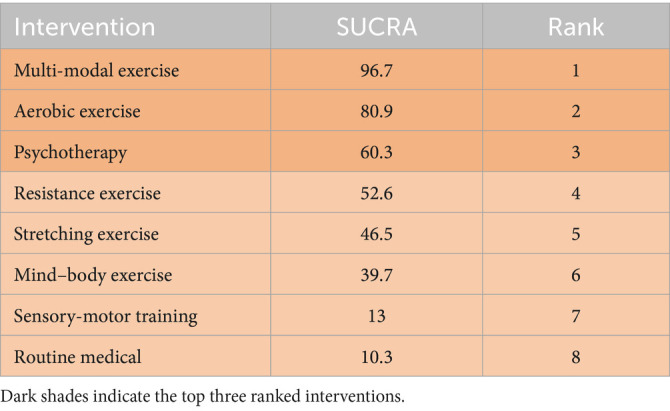

However, the effects of physical activity interventions on stroke patients remain controversial (29, 74), which may be due to differences in physical activity protocols and baseline physical conditions across different patient populations. Previous stroke rehabilitation studies have shown that physical activity improves cognitive function by altering protein integrity and activating key brain regions involved in cognitive processes (75). It has been observed in relevant studies that physical activity promotes neurogenesis, cell survival, synaptogenesis, synaptic plasticity, and angiogenesis processes associated with improvements in cognitive function (76). Cognitive impairments following a stroke are commonly characterized by an increased attention to negative stimuli, Challenges associated with disengaging from negative emotions often stem from deficits in cognitive control when processing such information (77). This study expands on existing knowledge by investigating how different types of physical activity interventions-specifically multi-modal exercise, aerobic exercise, and psychotherapy—affect cognitive function in stroke survivors. Recent research underscores the importance of both physical activity and mental stimulation in preserving brain health and slowing the progression of cognitive decline. Among these interventions, multi-modal exercise, which incorporates diverse types of physical activity, seems especially advantageous for improving cognitive recovery in stroke patients.

Several studies have provided recent evidence that multi-modal exercise yields more effective results compared to traditional single mode exercise (78, 79). This is likely attributed to the comprehensive stimulation it provides to the brain, including the enhancement of BDNF levels and improvements in cerebral blood flow dynamics. Unlike traditional physical activity interventions that focus on a single modality, such as aerobic or resistance training alone, multi-modal exercise provides a more comprehensive approach to rehabilitation. Multi-modal exercise has been demonstrated to substantially enhance cognitive function in individuals who have suffered from strokes, enhancing attention, language fluency, and logical memory, while simultaneously stimulating the brain, increasing BDNF levels, and improving cerebral blood flow (80, 81). Additionally, multi-modal exercise has shown more effective results than single mode exercise in enhancing overall cognitive function in patients with mild cognitive impairment (82). This is attributed to its comprehensive stimulating effect on the brain.

The benefits of aerobic exercise in cognitive recovery following stroke are multifaceted. These advantages are not only based on its direct benefits to the brain but also involve improvements in the overall health status of the patient (55, 56, 72). Aerobic exercise enhances cardiovascular health, which in turn indirectly promotes brain health by ensuring that the brain receives sufficient oxygen and nutrients, a crucial factor for maintaining and restoring cognitive function. Studies have shown that aerobic exercise increases the pumping capacity of the heart and improves blood circulation efficiency, thereby increasing cerebral blood flow. This is particularly effective for brain repair and functional recovery after stroke (83). Aerobic exercise is versatile in form and can be adjusted according to the patient’s abilities and stage of rehabilitation, making it easily implementable in both home and community settings (84). This flexibility makes it a cost-effective rehabilitation option. In addition, aerobic exercise helps improve the emotional state of stroke patients by alleviating symptoms of depression and anxiety, which is crucial for enhancing overall quality of life and motivation for recovery. A systematic review and meta-analysis by Li et al. (37) also demonstrated that aerobic exercise significantly improves cognitive function in stroke patients, including attention, memory, and executive function (85).

Additionally, resistance exercise has emerged as a targeted intervention in the rehabilitation of stroke patients, focusing on the recovery of muscle strength and function (49, 52, 62). The advantage of this form of exercise lies in its ability to directly target muscles, enhancing muscle strength and improving neuromuscular efficiency, which is crucial for functional recovery in post-stroke patients (86). Research has shown that resistance exercise can improve muscle strength and endurance in stroke patients, thereby increasing their ability to perform activities of daily living. This improvement may, in turn, indirectly promote the enhancement of cognitive function (5). Resistance exercise may have a positive impact on the fall risk in stroke patients by enhancing muscle strength and improving body balance, thereby reducing the risk of secondary injuries resulting from falls. This is of significant importance for improving patients’ quality of life and boosting their confidence. Research has demonstrated that stroke survivors who underwent a 12 week resistance exercise program showed significant improvements in lower limb strength and walking speed (37). Resistance exercise can also indirectly stimulate brain function by increasing blood flow and metabolic activity in muscles, particularly in brain regions associated with motor control. This stimulation may help promote neuroplasticity and improve cognitive function. In one study, stroke patients who underwent 8 weeks of resistance exercise showed improvements in cognitive function test scores, particularly in executive function and processing speed (26). As part of a stroke rehabilitation program, resistance exercise not only enhances muscle strength and improves physical function but also has a positive impact on cognitive function. Therefore, resistance exercise should be considered a crucial component of a comprehensive rehabilitation plan for stroke patients, contributing to overall rehabilitation outcomes and enhancing quality of life.

Complementing these statistical findings, visual aids such as Figure 3 (network diagram), Figure 4 (SUCRA plot), and Figure 5 (funnel plot) provide critical contextual insights that strengthen the interpretation of results. Figure 3, as a network diagram, illustrates the density of evidence across interventions: multi-modal exercise, aerobic exercise, and routine medical care emerge as central nodes with robust direct comparisons, while weaker connections (e.g., resistance exercise vs. sensory-motor training) rely primarily on indirect evidence, highlighting gaps where direct head-to-head trials are needed. Figure 4 (SUCRA plot) visually reinforces the ranking of interventions, with multi-modal exercise (96.7%) and aerobic exercise (80.9%) occupying the top positions, offering an intuitive representation of their relative effectiveness. Meanwhile, Figure 5 (funnel plot) supports the robustness of findings through its symmetrical distribution, indicating minimal publication bias and enhancing confidence in the overall results.

In conclusion, diverse modalities of physical activity interventions exhibit a favorable effect on cognitive function among individuals who have suffered a stroke. This research, conducted through a systematic review and network meta-analysis, substantiates the advantageous impacts of various physical activity interventions on cognitive abilities in stroke patients, aligning with the results reported in the current body of literature. These interventions support cognitive recovery by promoting neuroplasticity, improving cerebral blood circulation, and reducing inflammatory responses, among other mechanisms. Notably, multi-modal exercise and aerobic exercise have shown more effective results compared to traditional single type exercises, while resistance exercise, yoga, and Tai Chi have also demonstrated significant benefits in long term interventions. However, the study lacks critical assessment of heterogeneity among the 26 included studies—though I^2^ statistics were used to evaluate heterogeneity, the potential influences from varying intervention protocols (such as differences in exercise intensity, frequency, and duration across studies) and participant baseline characteristics were not thoroughly explored. Additionally, despite covering 12 countries globally, the research did not investigate possible cultural or regional differences, such as how cultural preferences for certain exercises (like Tai Chi in East Asia vs. aerobic exercise in Western regions) might affect outcomes. Therefore, in clinical practice, it is essential to consider individual patient characteristics and the duration of interventions when selecting the most appropriate exercise intervention to optimize the recovery of cognitive function in stroke patients.

## 5 Strengths and limitations

This study showcases several significant strengths. Firstly, it represents the first network meta-analysis aimed at exploring the impact of physical activity on cognitive function specifically in individuals who have experienced a stroke. This research provides valuable scientific insights that can guide the selection of suitable physical activity interventions designed to enhance cognitive function in this demographic. Furthermore, the incorporation of numerous studies significantly bolsters the reliability and precision of the findings. Thirdly, the robustness of the findings is further strengthened by the exclusive emphasis on randomized controlled trials, while intentionally excluding observational and cross-sectional studies. However, there are some limitations to consider. For instance, individual differences among stroke patients may result in varied responses to physical activity interventions, and the specific intensity or duration of physical activity may influence the overall effectiveness of these interventions.

Future research could concentrate on several important areas. First, investigating personalized physical activity interventions that are tailored to the unique characteristics of stroke patients could prove beneficial. For instance, factors such as the patient’s age, medical history, and the severity of post-stroke conditions (e.g., varying levels of depression or other comorbidities) should be taken into account when selecting appropriate interventions. Customizing physical activity regimens according to these individual variables may enhance their effectiveness in promoting cognitive recovery. Second, further studies examining the optimal parameters of physical activity—such as frequency, duration, and intensity—would be crucial for fine tuning intervention strategies and maximizing their potential to improve cognitive function in stroke patients.

## 6 Conclusion

This study examined the effects of various forms of physical activity on cognitive function in stroke patients. The results indicate that multi-modal exercise and aerobic exercise exhibit superior efficacy in promoting cognitive recovery, supported by their high SUCRA rankings (96.7 and 80.9%, respectively).

Furthermore, prior to implementing physical activity interventions, a thorough assessment of the individual characteristics of stroke patients is crucial to ensure the selection of the most appropriate type of physical activity and proper intervention dosage. Additionally, healthcare providers, whether in community or hospital settings, should engage in continuous communication with stroke patients, offering guidance and encouragement to help them adhere to consistent physical activity regimens, while also providing support to address any negative emotional challenges. In terms of future research, exploring personalized physical activity interventions tailored to the unique needs of stroke patients could be valuable. Moreover, further studies should examine the optimal dosage of various physical activity interventions to enhance cognitive function in stroke patients, aiming to improve the effectiveness and accuracy of these interventions.

Given the limitations of network meta-analysis, which primarily relies on indirect comparisons, future research should prioritize randomized controlled trials that directly compare top-ranked interventions (such as multi-modal exercise versus aerobic exercise) to validate their relative effectiveness. Additionally, long-term follow-up studies are needed to explore the sustained impact of these interventions on cognitive outcomes, as the current evidence is mainly based on short-to-moderate intervention durations (an average of 16 weeks). These efforts will help refine intervention strategies and improve the accuracy of clinical recommendations for post-stroke cognitive rehabilitation.

## Data Availability

The original contributions presented in the study are included in the article/Supplementary material, further inquiries can be directed to the corresponding authors.

## Author’s note

We hereby declare that the results of this study are presented clearly and honestly, with all data being true and reliable, and that no fabrication, falsification, or inappropriate data manipulation has occurred. Furthermore, the findings of this study represent the independent conclusions of the research team and do not reflect any endorsement or position of the American College of Sports Medicine (ACSM).
