# Supplementary material for: Physical activity interventions for post-stroke cognitive recovery: a systematic review and network meta-analysis of comparative effects
Source: Front Neurol. 2025 Sep 1;16:1646328. doi: 10.3389/fneur.2025.1646328 (PMC12434964; doi:10.3389/fneur.2025.1646328)

Physical Activity Interventions for Post-Stroke Cognitive Recovery: A Systematic Review and Network Meta-Analysis of Comparative Effects

Contents

Appendix A Search strategy..2

Appendix A1 Initial search strategy..2

Appendix B Publication Bias Test..8

Appendix B1 Results of Begg's and Egger's tests..8

Appendix B2 Begg's funnel plot..9

Appendix B3 Trim-and-fill plot..10

Appendix B4 Results of trim-and-fill analysis..11

Appendix B5 Sensitivity analysis table..12

Appendix B6 Sensitivity analysis plot..13

Appendix A Search strategy

Appendix A1 Initial search strategy

Database	Search strategy	amount
	PubMed	
#1	Search: **Exercises[MeSH Terms]**	293,471
#2	Search: **((((((((((((((Exercises) OR (Physical Activity)) OR (Activities, Physical)) OR (Physical Activities)) OR (Exercise, Physical)) OR (Physical Exercise)) OR (Acute Exercise)) OR (Exercise, Isometric)) OR (Isometric Exercise)) OR (Training, Exercise)) OR (motion)) OR (movement)) OR (athletic)) OR (sports)) OR (sport)) OR (motional)**	1,996,417
#3	**#1 OR #2**	1,996,417
#4	Search: **Stroke[MeSH Terms]**	181,337
#5	Search:**(((((((((((Stroke) OR (Cerebrovascular Accident)) OR (Cerebrovascular Accidents)) OR (CVA(Cerebrovascular Accident))) OR (CVAs (Cerebrovascular Accident))) OR (Cerebrovascular Apoplexy)) OR (Apoplexy, Cerebrovascular)) OR (Cerebrovascular Stroke)) OR (Apoplexy)) OR (Cerebrovascular Accident)) OR (Acute Cerebrovascular Accident)) OR (Cerebrovascular Accidents, Acute)**	468,391

#6	**#4 OR #5**	468,391
#7	Search:**Cognitive Function[MeSH Terms]**	207,623
#8	Search:**(((((((Cognitive Function) OR (Attention)) OR (Orientation)) OR (Memory)) OR (Executive Functions)) OR (Language)) OR (Social Cognition)) OR (Visuospatial)**	1,997,768
#9	**#7 OR #8**	1,997,768
#10	Search: **Randomized controlled trial[MeSH Terms]**	173,663
#11	Search: **((((Randomized controlled trial) OR (controlled clinical trial)) OR (randomized)) OR (placebo)) OR (randomly)**	1,908,543
#12	**#10 OR #11**	1,908,543
#13	**#3 And #6 And #9 And #12**	1,413

Embase		
#1	**'exercise'/exp OR 'activities, physical' OR 'physical activity' OR 'physical activities ' OR 'exercise, physical' OR 'physical exercise' OR 'acute exercise' OR 'exercise, isometric' OR 'isometric exercise' OR 'training, exercise' OR 'motion' OR 'athletic' OR 'motional'**	1,129,718
#2	**'stroke'/exp OR 'cerebrovascular accidents' OR 'cva(cerebrovascular accident)' OR 'cvas (cerebrovascular accident)' OR 'cerebrovascular apoplexy' OR 'apoplexy, cerebrovascular' OR 'cerebrovascular stroke' OR 'apoplexy' OR 'cerebrovascular accident' OR 'acute cerebrovascular accident' OR 'cerebrovascular accidents, acute'**	491,347
#3	**'randomized controlled trial'/exp OR 'controlled clinical trial' OR 'randomized' OR 'placebo' OR 'randomly'**	2,250,537
#4	**'cognitive function'/exp OR 'attention' OR 'orientation' OR 'memory' OR 'executive functions' OR 'language' OR 'social cognition' OR 'visuospatial'**	4,224,267
#5	**#1 And #2 And #3 And #4**	1,162

Web of Science		
#1	**(((((((((((((((TS=(Exercises)) OR TS=(Physical Activity)) OR TS=(Activities, Physical)) OR**	7,200,063

	**TS=(Physical Activities)) OR TS=(Exercise, Physical)) OR TS=(Physical Exercise)) OR TS=(Acute Exercise)) OR TS=(Exercise, Isometric)) OR TS=(Isometric Exercise)) OR TS=(Training, Exercise)) OR TS=(motion))) OR TS=(movement)) OR TS=(athletic)) OR TS=(sports)) OR TS=(sport)) OR TS=(motional) and Preprint Citation Index (Exclude – Database)**	
#2	**((((((((((TS=(Stroke)) OR TS=(Cerebrovascular Accident)) OR TS=(Cerebrovascular Accidents)) OR TS=(CVA(Cerebrovascular Accident))) OR TS=(CVAs (Cerebrovascular Accident))) OR TS=(Cerebrovascular Apoplexy)) OR TS=(Apoplexy, Cerebrovascular)) OR TS=(Cerebrovascular Stroke)) OR TS=(Apoplexy)) OR TS=(Cerebrovascular Accident)) OR TS=(Acute Cerebrovascular Accident)) OR TS=(Cerebrovascular Accidents, Acute)**	820,593
#3	**((((TS=(Randomized controlled trial)) OR TS=(controlled clinical trial)) OR TS=(randomized)) OR TS=(placebo)) OR TS=(randomly)**	2,370,330
#4	**(((((((TS=(Cognitive Function)) OR TS=(Attention)) OR TS=(Orientation)) OR TS=(Memory)) OR TS=(Executive Functions)) OR TS=(Language)) OR TS=(Social Cognition)) OR TS=(Visuospatial)**	9,308,705
#5	**#1 AND #2 And #3 And #4**	1,379

Cochrane		
#1	MeSH descriptor: **[Exercise] explode all trees**	38714

#2	**(Exercises) OR (Physical Activity) OR (Activities, Physical) OR (Physical Activities) OR (Exercise, Physical)**	124178
#3	**(Physical Exercise) OR (Acute Exercise) OR (Exercise, Isometric) OR (Isometric Exercise) OR (Training, Exercise)**	92923
#4	**(athletic)**	7123
#5	**#1 OR #2 OR #3 OR #4**	159773
#6	MeSH descriptor: **[Stroke] explode all trees**	17473
#7	**(Stroke) OR (Cerebrovascular Accident) OR (Cerebrovascular Accidents) OR (CVA(Cerebrovascular Accident)) OR (CVAs (Cerebrovascular Accident))**	90657
#8	**(Cerebrovascular Apoplexy) OR (Apoplexy, Cerebrovascular) OR (Cerebrovascular Stroke) OR (Apoplexy) OR (Cerebrovascular Accident)**	25608
#9	**(Acute Cerebrovascular Accident) OR (Cerebrovascular Accidents, Acute)**	5095
#10	**#7 OR #8 OR #9 OR #10**	91154
#11	**MeSH descriptor: [Randomized Controlled Trial] explode all trees**	37
#12	**(Randomized controlled trial) OR (controlled clinical trial) OR (randomized) OR (placebo) OR (randomly)**	1638751
#13	**#12 OR #13**	1638751
#14	MeSH descriptor: **[Cognition] explode all trees**	16241

#15	**(Cognitive Function) OR (Attention) OR (Orientation) OR (Memory) OR (Executive Functions)**	91181
#16	**(Language) OR (Social Cognition) OR (Visuospatial)**	2156256
#17	**#14 OR #15 OR #16**	2156515
#18	**#5 And #10 And #13 And #17**	8094

Appendix B Publication Bias Test

Appendix B1 Results of Begg's and Egger's tests

```
Begg's Test

  adj. Kendall's Score (P-Q) =    -155
          Std. Dev. of Score =   79.54 (corrected for ties)
           Number of Studies =      38
                           z =   -1.95
                    Pr > |z| =   0.051
                           z =    1.94 (continuity corrected)
                    Pr > |z| =   0.053 (continuity corrected)

Egger's test
```

Std_Eff	Coef.	Std. Err.	t	P>|t|	[95% Conf.	Interval]
slope	2.551707	.1008417	25.30	0.000	2.34719	2.756223
bias	-.1589655	.8407926	-0.19	0.851	-1.864172	1.546241

Appendix B2 Begg's funnel plot

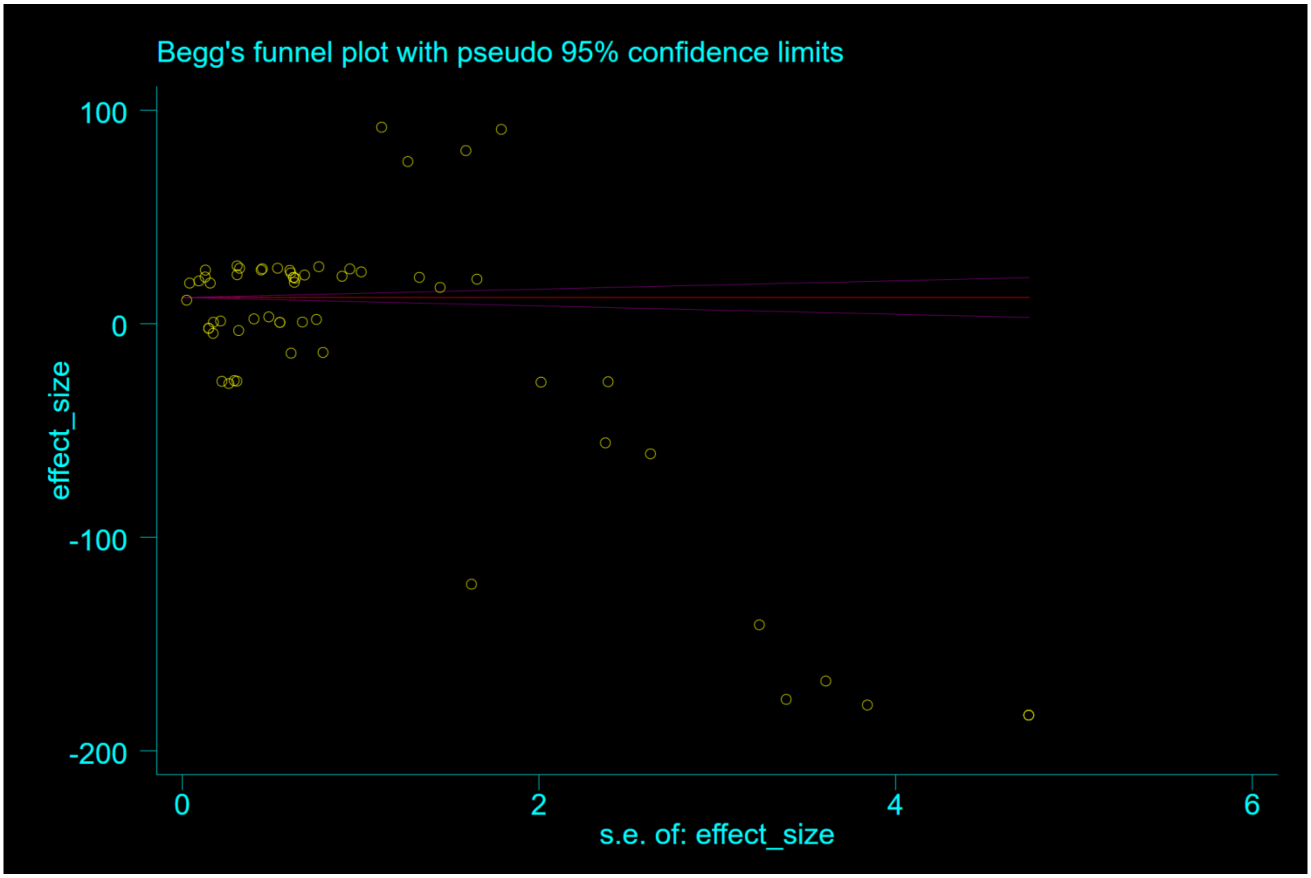

Appendix B3 Trim-and-fill plot

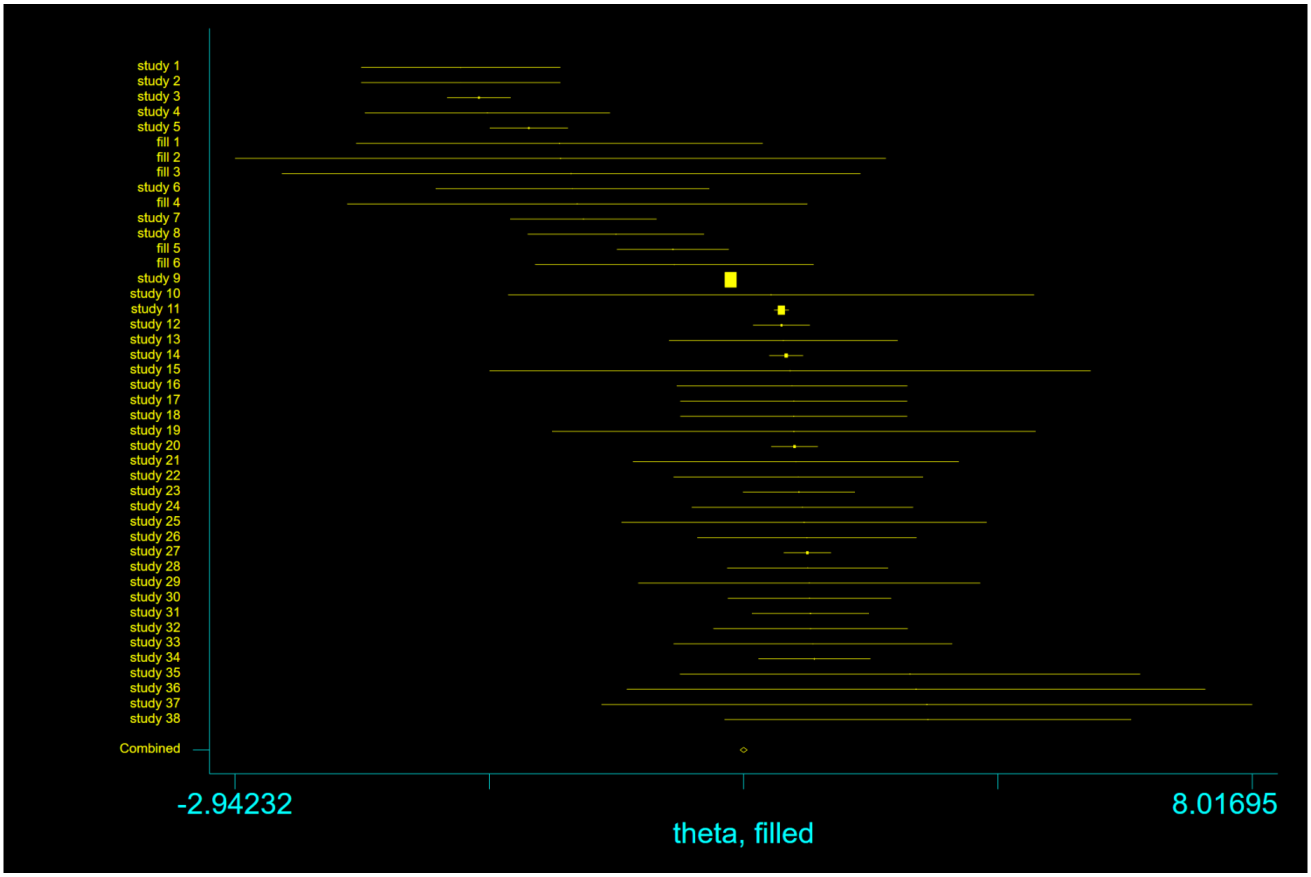

Appendix B4 Results of trim-and-fill analysis

Model	Before	After
Fixed-effects model	2.542 (2.504 - 2.581)	2.537 (2.499 - 2.575)
Random-effects model	2.420 (2.136 - 2.703)	2.329 (2.058 - 2.600)

Appendix B5 Sensitivity analysis table

Study omitted	Estimate	[95% Conf.	Interval]
1	2.5423748	2.5042412	2.5805085
2	2.5423748	2.5042412	2.5805085
3	2.5267687	2.4882011	2.5653362
4	2.5296242	2.4910457	2.5682027
5	2.5423748	2.5042412	2.5805085
6	2.5423748	2.5042412	2.5805085
7	2.5423748	2.5042412	2.5805085
8	2.5423748	2.5042412	2.5805085
9	2.539758	2.5015545	2.5779612
10	2.540009	2.5017991	2.5782189
11	2.5446031	2.5064387	2.5827675
12	2.5464301	2.5082519	2.5846083
13	2.5423748	2.5042412	2.5805085
14	2.5423748	2.5042412	2.5805085
15	2.5423748	2.5042412	2.5805085
16	2.5423748	2.5042412	2.5805085
17	2.5421321	2.5039914	2.5802729
18	2.5422602	2.5041225	2.5803978
19	2.5414255	2.5032666	2.5795844
20	2.5416698	2.5035164	2.5798235
21	2.5217576	2.4827666	2.5607488
22	2.5360658	2.4976342	2.5744975
23	2.5393388	2.5011289	2.5775487
24	2.5417325	2.5035796	2.5798857
25	2.5421116	2.5039692	2.5802543
26	2.5419695	2.5038178	2.5801215
27	2.541889	2.5037374	2.5800405
28	2.5418544	2.5037024	2.5800066
29	2.5419044	2.5037556	2.5800533
30	2.5418544	2.5037024	2.5800066
31	2.5418966	2.5037506	2.5800424
32	2.5420742	2.5039325	2.5802162
33	2.5410507	2.5028811	2.5792201
34	2.5410504	2.5028799	2.5792208
35	2.5423748	2.5042412	2.5805085

36	2.5423748	2.5042412	2.5805085
37	2.5446811	2.5065317	2.5828307
38	2.5462301	2.5080724	2.5843878
Combined	2.5423748	2.5042412	2.5805085

Appendix B6 Sensitivity analysis plot

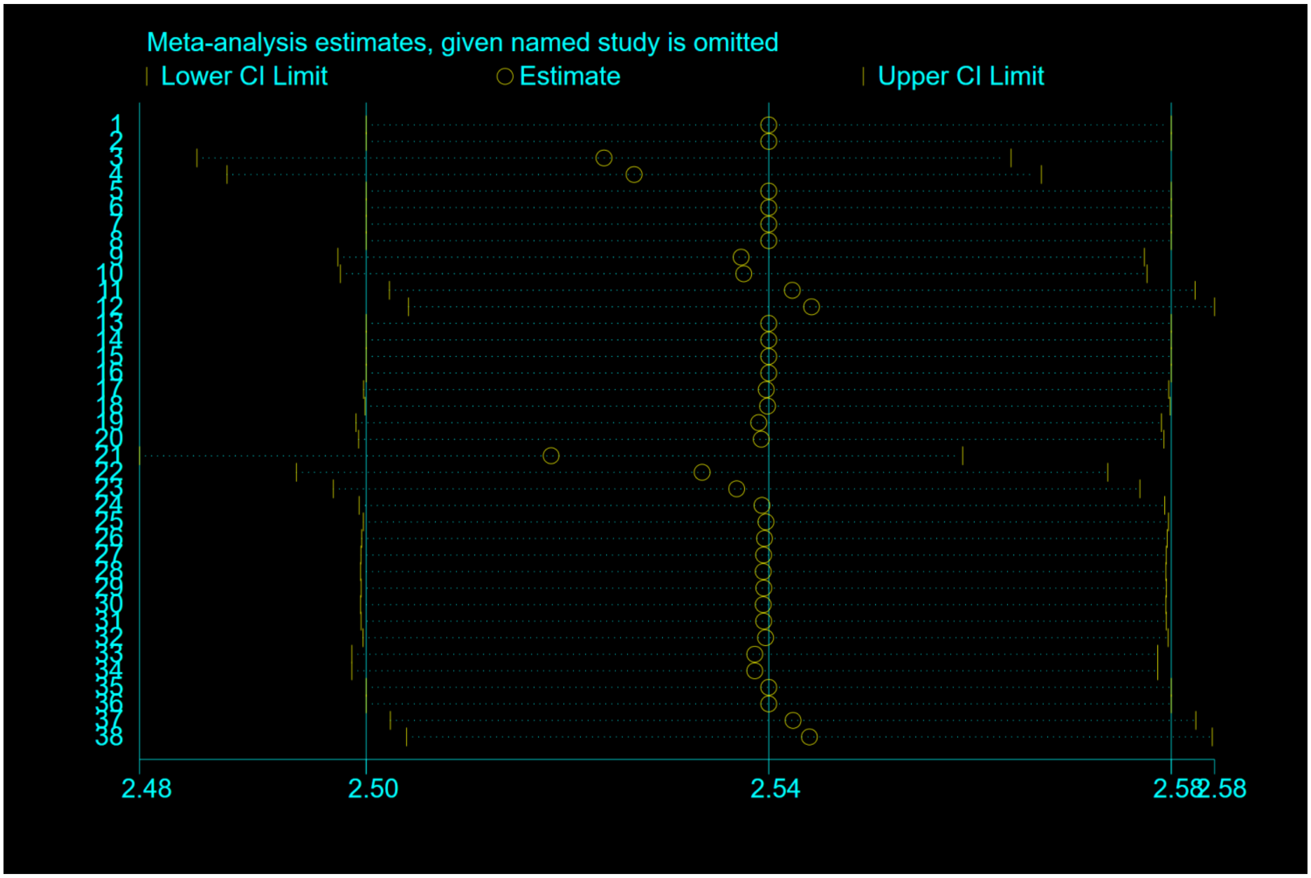

Supplement: Supplementary file 1 [file Data_Sheet_1.pdf]
